# Dental Management of a Child with Incidentally Detected Hemophilia: Report of a Clinical Case

**DOI:** 10.1155/2017/7429738

**Published:** 2017-05-28

**Authors:** Ricardo Martínez-Rider, Arturo Garrocho-Rangel, Raúl Márquez-Preciado, María Victoria Bolaños-Carmona, Socorro Islas-Ruiz, Amaury Pozos-Guillén

**Affiliations:** ^1^Pediatric Dentistry Postgraduate Program, Faculty of Dentistry, San Luis Potosi University, 78290 San Luis Potosí, SLP, Mexico; ^2^Facultad de Odontología, Universidad de Granada, Campus Universitario de Cartuja, 18071 Granada, Spain

## Abstract

Children with hemophilia (A or B) are at risk for bleeding episodes, which rank from mild mucosal/soft tissues bleeding to life-threatening hemorrhages. This report describes the dental/medical management provided to an 8.10-year-old patient suffering from uncontrolled bleeding after a surgical procedure to expose both permanent upper central incisors, in which hemophilia was a pure incidental finding. Additionally, diverse precautions to be considered during the dental clinical treatment of hemophilic children are discussed.

## 1. Introduction

Hemostasis disorders are classified as coagulation factor deficiencies, platelet disorders, vascular disorders, and fibrinolytic defects [1]. Hemophilia belongs to the first group of these diseases, clinically characterized by prolonged clotting time and excessive bleeding into mucosa, soft tissues, muscles, and weight-bearing joints [1, 2]; joint bleeding or* hemarthroses *can result in debilitating arthropathy [3]. It has been associated with mortality and morbidity and therefore with numerous impacts on overall health [3–5]. In hemophilia, some of these clotting factors are abnormal, in number or structure [6]. Consequently, the blood clotting cascade may be disturbed, resulting in a significant increased bleeding time, evidenced mainly by a prolonged activated partial thromboplastin time (aPTT) [5, 7].

Hemophilia is an X chromosome-linked hereditary lifelong bleeding disorder, with a frequency of 1 in 5,000–30,000 births, approximately [3, 8]. This disease has been subclassified in three subtypes:* A*, representing 80–90% of total cases (or 1 : 5000 births), where females are carriers, males are only affected, and male-to-male transmission does not occur;* B* (Christmas disease), which is much less common (1 : 30,000 births); and* C* (Rosenthal syndrome), also very rare [3, 8]. Additionally, a fourth type of hemophilia was proposed by the Norwegian physician Owren in 1947 [9], the* Owren's disease* or* parahemophilia*, caused by a deficient factor V, with an incidence of 1 case per 1 million children [1, 10].* A* and* B* types are clinically indistinguishable [3] and are caused by deficiencies of the coagulation mechanism factors VIII (or antihemophilic factor) and IX (or plasma thromboplastin component), respectively;* C* subtype results from a deficiency of factor XI [2, 8]. There is no specific racial or geographic preference for the disease. Although it is passed down from parents to children, the disorder shows no familiar history; about 1/3 of cases are caused by a spontaneous or sporadic mutation [11, 12]. The prognosis of affected children depends on their incapacity degree, presence of antibodies against factor VIII, and presence of hepatitis or other liver diseases, or HIV/AIDS [6].

Child oral health care, even invasive procedures, can be delivered in a safe manner in the dental office, as long as diverse measurements and precautions are properly instituted [6, 7, 9, 13]. The purposes of the present report are to describe and discuss the diagnostic and therapeutic actions provided to a pediatric patient suffering a minor craniofacial trauma, in which hemophilia was a pure incidental finding.

## 2. Case Report

**Figure 1:**
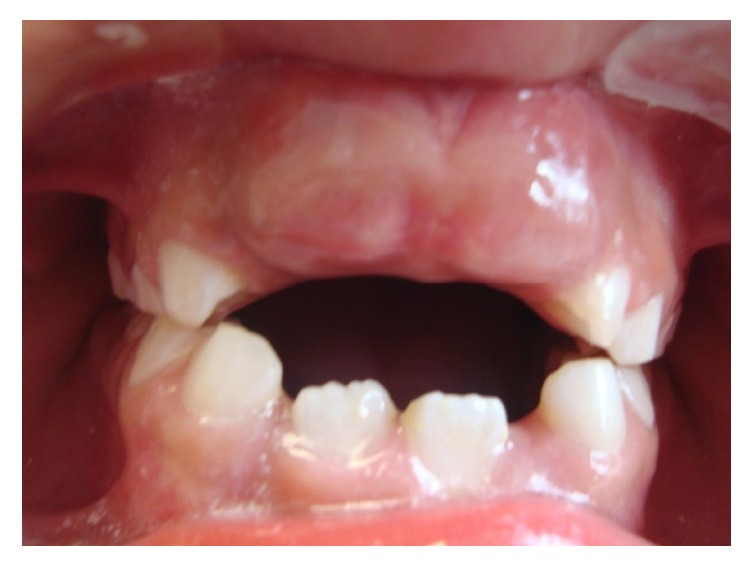
Initial frontal view.

An 8.10-year-old boy without history of significant bleeding events presented with his parents to the Pediatric Dentistry Postgraduate Clinic complaining of lack of eruption of both permanent upper central incisors. The parents manifested a good general health status of their son and no reported previous significant bleeding episodes (e.g., from gingiva during tooth brushing), medical disorders (particularly bleeding diathesis), or exposure to surgical interventions. On the oral examination, both incisors were palpable and covered with a fibrous gingival tissue, not associated with previous bleeding (Figure 1).

**Figure 2:**
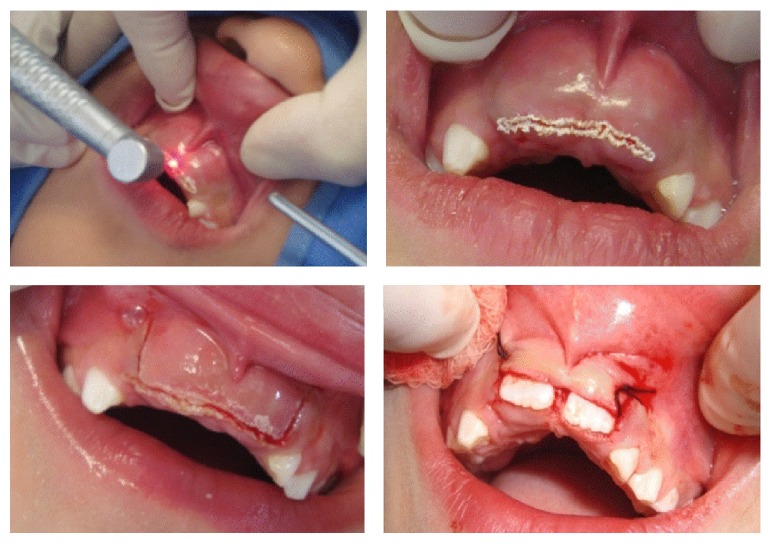
Surgical procedure stages.

A signed informed consent was obtained from the parents before the treatment. It was decided to perform a vestibular squared incision over the gingiva with flap apical reposition, to expose the incisal third of both incisor crowns. The surgical procedure was carried under local anesthesia, employing a water-irrigated laser hand-piece system (Waterlase YSGG®, Biolase Technology, Inc., Irving, CA, USA) and sutures in both sides of the flap (Figure 2). The patient was discharged without apparent local or systemic complications and with postoperative hygiene/diet instructions.

**Figure 3:**
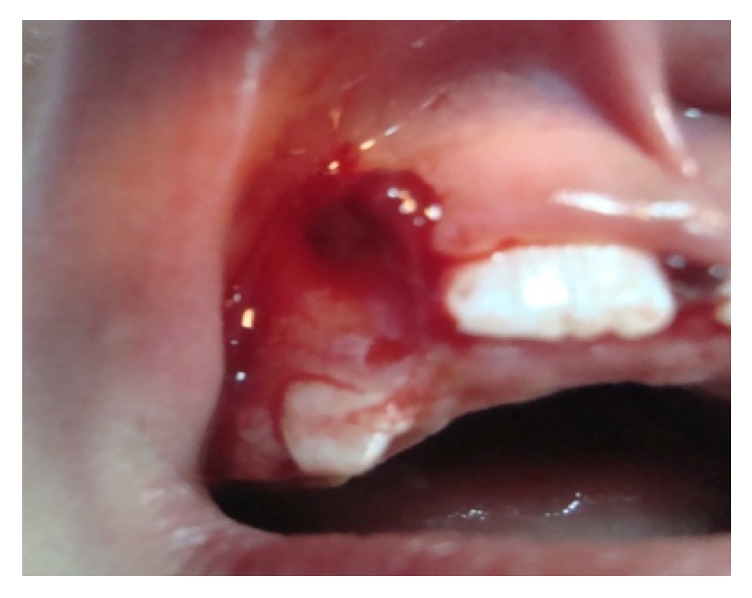
3-day postoperative view. The right suture was removed. The wound was resutured.

**Figure 4:**
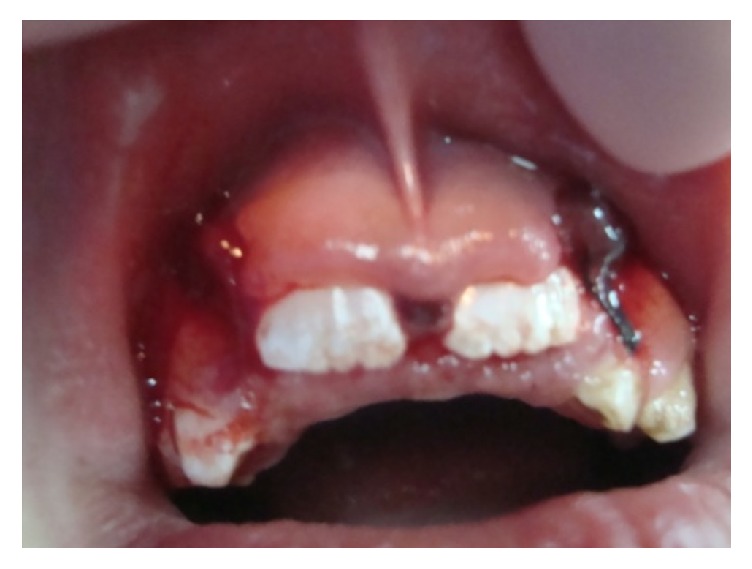
2 days later. The new suture was also retired and then resutured. Pharmacological treatment was initiated intravenously.

**Figure 5:**
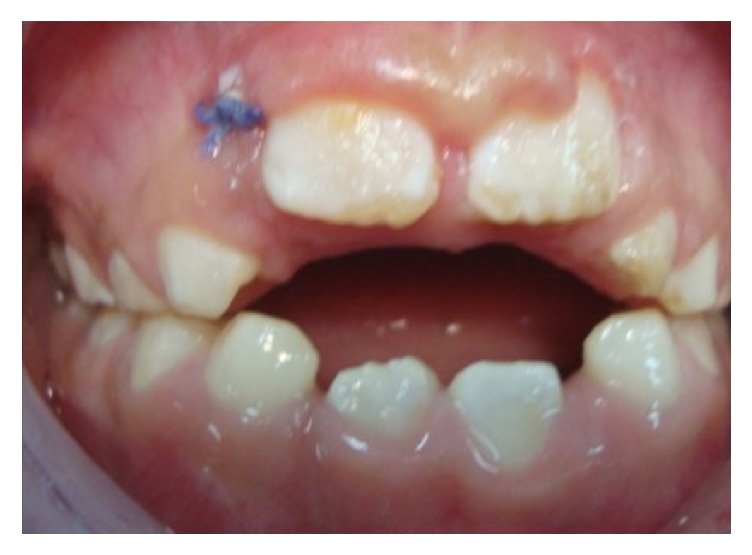
Final view after 8 hours of treatment. Bleeding was finally controlled.

Three days later the patient returned to the clinic exhibiting gingival profuse bleeding, difficult to control with external pressure application by means of wet gauze (Figure 3). After consulting with the Pediatric Hematologist and because there was not history of spontaneous bleeding or other hemorrhagic events, routine laboratory blood tests for blood clotting times were carried out. The results were within the normal limits, except for the aPTT, which was considered slightly lower. Then, the wound was resutured and covered with Gelfoam with a surgical splint. The patient was closely monitored in an ambulatory approach. After 2 days, the bleeding persisted (Figure 4) and the child looked pale and weak. New laboratory specific tests, including quantification of factors VIII and IX and Von Willebrand, were performed. The clot factor VIII manifested a deficit of 6% regarding the normal plasmatic level; according to this information, the child was diagnosed as having mild hemophilia A. To control the bleeding, the patient was intravenously infused with tranexamic acid (250 mg), vitamin K (5 mg), and normal saline, for 8-hours; after this time, the hemorrhage was finally stopped, and the patient discharged. Close control visits were programmed. At the final examination, 10 days after the event, the child did not show additional unusual bleeding episodes (Figure 5) or any other oral/systemic complications.

## 3. Discussion

Most guidelines for managing hemophilia and other bleeding disorders have been developed based on experiences in adults [3, 6, 7, 13–17]. Currently, no universally standard protocol specific for affected children is available, to ensure a safe treatment in the clinical setting [8, 12].

In hemophilic children, the highly vascular oral cavity is frequent for hemorrhage episodes. The most common sites of bleeding are the frenum of the lip and the tongue, after a procedure-induced trauma [7, 10, 18]. Gingival spontaneous bleeding is also frequent, due to minor stimuli such as tooth brushing, food abrasion or infection [19]. In these oral regions there are a significant number of enlarged capillaries near the surface. Therefore, the tendency of bleeding is higher [19], particularly as the child increases physical activity or under emotional stress associated with dental treatment [6].

Hemophilia A is also classified in relation to its harshness as severe, moderate, and mild [2, 4]. In severe cases, when there is less than 1% of factor VIII normal plasmatic level, which is 50 to 100 IU/dL, patients are prone to bleed spontaneously in cases of mouth lacerations, during the time of eruption or shedding of primary teeth and even without discernible trauma; in situation of oral needs, children should be managed in conjunction with a multidisciplinary medical team, preferably in a hospital owning a hemophilia treatment center. Children with moderate and mild hemophilia A (1 to 5% and 5 to 25% of normal plasmatic level, resp.) usually exhibit uncontrollable bleeding episodes secondary to a major trauma or surgery [2]; these patients may be treated in the dental clinic, in collaboration with a hematologist [5]. In many cases, mild hemophilia may not be diagnosed until adolescence or later, particularly when patients have not undergone to tooth extractions, major surgeries, or trauma during childhood [19].

When programming dental treatment, it is important that clinical practitioners know the half-life of the deficient clotting factor. Operative sessions should be comprised on consecutive days, if replacement therapies are administrated [1, 5, 6]. However, 10–30% of A hemophilic children develop antibodies against factor VIII as a result of replacement therapy, which give rise to significant difficulties [6, 8, 20]; in these cases, in accordance with the hematologist, the problem can be solved by administering antihemophilic factor A [6].

For dental invasive procedures, local homeostasis is improved by means of using antifibrinolytic agents as adjuvant therapies, in addition or instead of replacement therapy. Some authors [5, 6, 21, 22] have suggested the use of tranexamic acid, both topically and systemically, in order to reduce bleeding complications after a dental surgery. Topically, this drug is applied into the socket after a tooth extraction, and systemically, a dose of 1 g (or 30 mg/kg) is given preoperatively, orally, or in infusion. On the other hand, desmopressin (a synthetic analog of vasopressin) has been also successfully used as intravenous infusion or intranasal spray, in cases of mild/moderate hemophilia, prior to dental treatment; this vasoactive drug has demonstrated an up to 4.7-fold increase of factor VIII level in plasma, sufficient to allow a safe oral management [23].

Pediatric dentists play a significant role because she/he may be the first to suspect a diagnosis of hemophilia in a child. However, routine screening for hemostasis disorders is hardly ever considered in the clinical setting, because most children seeking for dental attention do not refer any previous episode of excessive bleeding; then, as in our patient, uncontrolled bleeding during or after an oral surgery may be an incidental finding for suspecting the presence of hemophilia [2].

Studies have reported that the prevalence of caries, in both the primary and permanent dentition, and gingivitis were lower in children with hemophilia than the healthy general population [10, 24, 25]. According to these studies, this may attributed to the fact that many hemophiliacs children, from a very early age, attended dental departments during their hematological control visit and received a more vigorous and continuous caries prevention program than the general population. Also, patients and their parents are better motivated for a better oral hygiene, by virtue of their medical condition. In addition, needed oral treatment is usually provided by an appropriate dental staff, who are very familiar with hemophilia in children, as an integral part of the medical review [10, 24, 25]. Therefore, professional dental plaque control, oral hygiene and nutrition education, and access to specialized oral health services are paramount to improve oral health of hemophilic children.

## 4. Conclusions

Pediatric dentists should always give special attention and be aware of the potential risks of bleeding disorders; hemophilia is the most common clotting disorder worldwide and represents a serious challenge during the clinical practice, since routine dental treatment can produce life-threatening conditions. In diagnosed children, it is imperative careful screening for clotting deficiencies, particularly in those who are undergoing oral invasive or surgical procedures. Also, professional dental plaque control, oral hygiene and nutritional education, and access to specialized oral health services are paramount to improve oral health of hemophilic children.
